# Participation in a Short-Term Socialization and Training Program Improved Kennel-Raised Dog Welfare

**DOI:** 10.3390/ani16030485

**Published:** 2026-02-04

**Authors:** Nancy H. Ing, Reagan Richardson, Tennille K. Lamon, Courtney L. Daigle

**Affiliations:** 1 Department of Animal Science, Texas A&M University, 2471 TAMU, College Station, TX 77843, USA; reagan.richardson@tamu.edu (R.R.); cdaigle@tamu.edu (C.L.D.); 2 Comparative Medicine Program, Texas A&M University, 4473 TAMU, College Station, TX 77843, USA; tennillek@tamu.edu

**Keywords:** animal welfare, canines, enrichment, human-animal interaction, stress relief

## Abstract

Kennel-raised dogs are exposed to more stressors and fewer positive experiences than dogs raised in homes. We created a Dog Socialization and Training class to enrich the environment of university teaching dogs. Undergraduate students (103 total) were assigned a dog (64 total) to socialize and train for at least 36 30 min periods across 12 weeks. Each student used a Qualitative Behavior Assessments (QBA) tool to score 20 different behaviors on a scale of 0 to 125 for his/her dog at the beginning (PRE) and the end (POST) of each of the nine semesters. The PRE QBA scores were high for the ten positive valence behaviors including Relaxed (mean ± SE: 75 ± 6) and low for the ten negative valence behaviors including Distressed (31 ± 3). Following the interactive experiences, QBA scores for all 20 behaviors appeared to improve from 9% to 53% (*p* < 0.006). Principal Component Analysis identified four dimensions in the QBA scores that were responsible for most of the data variance. Providing kennel-housed dogs with regular, positive human interactions was perceived to increase the dogs’ positive valence towards humans and reduced negative responses. These benefits are expected to enhance the dogs’ teaching effectiveness with veterinary students, adoptability, and future adaptation to new forever homes.

## Simple Summary

Kennel-raised dogs have limited experiences and are exposed to more stressors, so they are more prone to developing fear-based behaviors than dogs raised in a home. Other studies have shown that increased positive interactions with people benefit the welfare of kenneled dogs. We created our Dog Socialization and Training class for undergraduate students to enrich the environments of university teaching dogs. To assess effectiveness, each student rated his/her dog for 20 behaviors early and late in the semester. In between, they socialized and trained their dogs in 36 30 min sessions over 12 weeks. The early behavior ratings were favorable, demonstrating that the dogs were well cared for. Remarkably, after the socialization and training, the ratings for each of the 20 behaviors appeared to improve. The greatest changes were in Relaxed behavior, which increased by 53%, and Distressed behavior, which decreased by 50%. We conclude that the environmental enrichment with positive human interactions was associated with improvements in the dogs’ behavioral profile and these benefits may make them more successful in teaching veterinary students and in their adaptation to future adoptive homes. The strengthening of the dog–human bond can occur at any age and has the potential to improve dogs’ welfare.

## 1. Introduction

The welfare and behavior of teaching and research animals are critical to their effectiveness. More than 42,000 dogs were used for research and teaching in the United States in 2023 [1]. Dogs have physiological and genetic similarities to humans that make them superior models for many translational studies in biomedical research [2,3,4,5,6,7,8]. Institutionalized dogs are also used for teaching purposes, such as in training courses for veterinarians and veterinary technicians [9,10]. Management strategies that minimize distress improve both the welfare of the animals and the quality of the education and science produced.

Dogs used for teaching and research are traditionally raised and housed in kennels. Kennels are different environments compared to private homes: they are noisier and have smaller spaces and fewer diverse objects [11]. Kennel-raised dogs can have increased fear of new people, objects, and environments, and even decreased trainability [12,13,14]. In a recent study they conducted behavior tests on 90 bitches and their 390 puppies in commercial breeding kennels in the USA and the related dogs had similarities in qualitative behavior (isolation tests) and quantitative (fecal cortisol metabolites) assessments [15]. Dogs that, prior to being rescued, had experienced adversity during early life while in commercial breeding kennels or hoarding situations were reported, by their new owner, to perform more nonsocial fears (e.g., fear of loud noises or unfamiliar objects) compared to control dogs [16]. In general, being in a kennel can negatively impact the behavior of dogs, and these impacts can stay with the dog even after the dog changes environments.

Dogs housed in kennels benefit from animate (dog and human) and inanimate enrichment [17]. Some dogs benefit more from human socialization than from socializing with other dogs [17,18,19,20]. An English study determined that even short periods (30 s per day) of intensive handling by a human for two months benefited the behaviors of research beagles [21]. An Australian group found that shelter dogs that had two minutes of positive contact with a person per day over five days stayed near their person longer on the sixth day than did control dogs [22]. Hair concentrations of cortisol, reflecting chronic stress levels, were higher in dogs in a large shelter in the Netherlands compared to their values six weeks and six months after adoption by private owners [23]. A study of privately owned German shepherds in Sweden showed that positive interactions with people correlated with lower levels of cortisol in the dogs’ hair [24]. In commercial breeding kennels in the USA, hair cortisol concentrations were inversely correlated to the number of socialization practices employed by their kennel [25]. Overall, the need to improve kenneled dog welfare can be partially met by increasing positive interactions with people.

The purpose of our Dog Socialization and Training class was to benefit the welfare of teaching dogs on campus by having undergraduate students socialize and train them. Since the class initiated in 2018, students worked with the dogs on socialization and basic obedience for at least three 30 min sessions per week. To document improved behaviors in the dogs in the class, the students completed Qualitative Behavior Assessments (QBAs) at the beginning (PRE) and end (POST) of the semester. The QBA was originally developed and validated to assess behavior in pigs [26,27] and later applied to a range of farm and domestic animals, including dogs [28,29,30,31,32]. Now, QBAs have been validated by correlating to quantitative physiological measures including cortisol levels [33]. In the class QBA, there were ten positive valence and ten negative valence behaviors assessed, where “valence” is the emotional value of a stimulus, ranging from pleasant/positive to unpleasant/negative [34]. This generated >5000 data points assessing 20 behaviors each semester, making this study a robust evaluation of changes in the welfare status of the teaching dogs. To the authors’ knowledge, this is the first study characterizing the impact of a semester-long undergraduate-level canine socialization and training course on dog behavior and welfare.

## 2. Materials and Methods

### 2.1. Dogs and Housing

All the dogs were institutionally owned by Texas A&M University and the activities outlined in this paper were approved by the Institutional Animal Care and Use Committee (IACUC) with AUPs 2017-0408, 2020-0299, and 2023-0185. The dogs were also on a teaching protocol that was approved by the IACUC. The tenure of the dogs at Texas A&M University is usually three years, before being retired and adopted into private forever homes.

This study was conducted every fall and spring semester at Texas A&M University from Fall 2018 to Spring 2024 except for three semesters during the COVID-19 pandemic resulting in a total of 64 dogs participating. Thirty-three of the 64 total dogs in this study participated in multiple semesters: 12, 14, and 7 dogs were in the class for two, three, or four times, respectively. In most cases the semesters were consecutive, but with a different assigned student. The dogs were mostly (67% of the total) hounds and hound crosses, including coonhounds (redbone, black and tan, bluetick, and treeing walker) and bloodhounds. These were followed by golden retrievers and mixes (16%) and then by Labrador retrievers and crosses (15%). There was one German shepherd cross. The teaching colony primarily receives dogs from the Texas Department of Criminal Justice (TDCJ), which operates units all over the state of Texas, USA. TDCJ predominantly uses hounds for tracking and contraband surveillance. Dogs that are unable to complete the rigorous training program to become a TDCJ working dog are then available to other state agencies such as Texas A&M University. The dogs used in the socialization class are kennel-raised, working breeds generally known for intelligence and trainability.

The dogs weighed 27.4 ± 0.7 kg and were 3.4 ± 0.2 years old (means ± SEs) at the beginning of the semester. For the dogs that participated for more than one semester, their average age over those semesters was used. All dogs were fed according to their bodyweight. All dogs were spayed or neutered prior to enrollment in the study. Dogs were either solo housed in climate-controlled kennels measuring 2.5 m × 2.2 m × 2.5 m (L/W/H) or housed in pairs in kennels with twice that floor area [35]. Kennels were cleaned once in the morning and once in the afternoon on weekdays and once in the morning on weekends and holidays. Dogs were fed a veterinarian-assigned portion of dry food (Purina Pro Plan: either Weight Management (Chicken and Rice Formula) or Sensitive Skin and Stomach (Lamb and Oatmeal)) twice a day. Each dog was provided with a minimum of two toys 24 h a day. The facility is on five acres and is equipped with nine outdoor grassy play yards containing enrichment toys. In addition to their interactions with the students, the dogs were let off leash in the yards to exercise and play under staff supervision at least three times a week. Approximately once a month, dogs were used as teaching animals at the Texas A&M University College of Veterinary Medicine.

### 2.2. Dog Training

The dogs were assigned to a student by a senior staff member who was familiar with the dogs’ personality and behavior. The staff member met the students on the first day of class where they gave a brief background of their experience handling dogs, which varied from novice with no dog ownership or handling experience to a lifetime of personal dog ownership and significant experience with handling/training dogs in various settings. To limit selection bias, the dog assignments were only based on the student’s background and handling skills: calmer dogs with novice handlers and excited dogs with experienced handlers. Of the 103 students enrolled in the class during the nine semesters in this report, 14 participated for multiple semesters: nine for two semesters, four for three semesters, and one for five consecutive semesters. In all cases except one, different dogs were assigned each semester. The training methods used were positive reinforcement (e.g., clicking when the dog sits and treating with a high-value reward) and negative punishment (e.g., not letting a dog that is pulling on the leash go forward). The dogs got to select their desired treats from the following: Great Value sliced turkey breast; Zuke’s Mini Naturals (Beef, Chicken, or Salmon Recipes, or Peanut Butter and Oats flavor); either of the dry dog foods (listed above); Milkbone Minis; Great Value Creamy Peanut Butter; and canned Purina Pro Plan Complete Essential (Beef and Rice Entrée). Most dogs preferred the food treats roughly in the order listed, from highest to lowest preference. Students carried the treats in Treat Pouch Sport bags (PetSafe^®^, Knoxville, TN, USA). If the dog did not care for food treats, they worked for playing with toys like tennis balls or for petting and praise. Each dog was fitted for a Blue-9 Balance Harness^®^ Buckle Neck (Maquoketa, IA, USA). Students attached their five-foot long, flat biothane leashes (www.dogid.com, accessed on 10 June 2025) to the D-ring on the front of the dog’s chest. These front connecting harnesses were favored by the professional trainers assisting the class and were demonstrated to be effective at reducing pulling on the leash by dogs without being aversive [36]. Twenty-foot-long biothane leashes were also available for training dogs at greater distances, such as for coming. Several training spaces with varying distraction levels were available for students to choose from. One indoor choice was one of two quiet exam rooms in a clinic. Another indoor training option was an empty kennel building, identical to those used to house the dogs. In addition, students could do their dog’s training outdoors in the fenced play yards (small, medium, and large) or while leash walking on cement sidewalks, the gravel parking area, or open grassy areas between the buildings. The dog training goals chosen for the Dog Socialization and Training class were loose-leash walking, ascending and descending stairs, sit, down, stay, come, touch, spin, and paws up. Loose-leash walking was prioritized because the dogs had been allowed to pull on leashes previously. In addition, loose-leash walking is highly desired by people adopting the dogs. The class employed two senior professional dog trainers from Puppy Love Dog Training (College Station, TX, USA) to provide expert advice and demonstrate training techniques to the student–dog pairs during ten-minute meetings held every two weeks.

**Figure 1:**
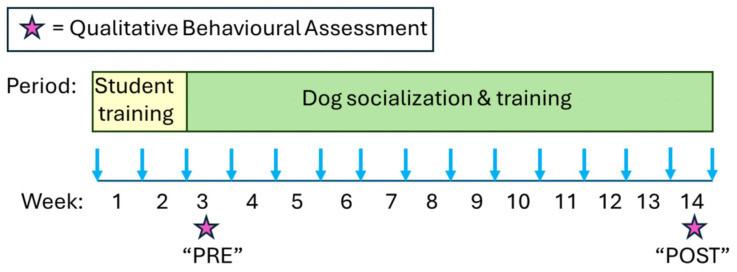
Timeline of student activities in a semester. In the first two weeks, the students learn to train dogs and they perform the required compliance assignments and facility orientations. In each of the following 12 weeks, every student socializes and trains their assigned dog for at least three 30 min sessions. The students conduct Qualitative Behavior Assessments (QBAs) during week 3 (“PRE” values) and 14 (“POST” values).

Undergraduate students of all levels responded to class flyers and email advertisements for this Directed Studies ANSC 291/495 class. Most students were Animal Science majors in the home department of the authors. In the first two weeks of the semester, students did safety training, completed course and facility orientations, and learned to use clicker training and positive reinforcement to socialize and train dogs (Figure 1).

The hand and voice cues for the behaviors were standardized and posted for consistency for the dogs and their students and care staff. In the final 12 weeks, each student was assigned to work with one dog on socialization and training. Students were required to visit and work with their dogs for a minimum of thirty minutes at least three days in each of the 12 weeks except in cases of severe weather, university holidays, or student illness.

Communication was critical to the success of the Dog Socialization and Training class. The goals for the training of the dogs at Texas A&M University with verbal and hand signal cues were decided on by the animal care staff and the consulting professional dog trainers to ensure that training was consistent across dogs and semesters. At the end of each semester, the students wrote a Dog Information Sheet about their assigned dog that included a cue chart. These provided continuity in the training methods used by the care staff members between semesters, students who were assigned to continue the dog’s training in the next semester, and eventually by the new owners of the adopted dog. All these communications helped coordinate training efforts to optimally benefit each dog.

### 2.3. Qualitative Behavior Assessments (QBAs)

Qualitative Behavior Assessments (QBAs) were done for 20 different behaviors. The QBAs were developed by a consortium [37] and were validated by quantitative measures [38,39]. Operational definitions of each QBA metric are presented in Table 1.

**Figure 2:**
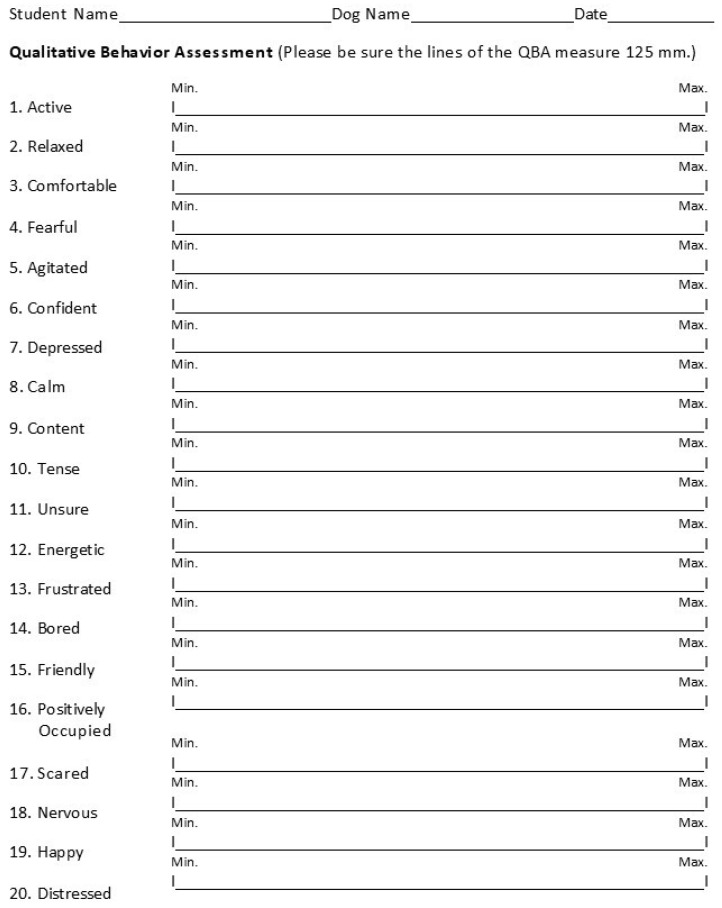
The Qualitative Behavior Assessment (QBA) form used by the students to assess 20 different behaviors on a 0 (“Min”) to 125 mm (“Max”) visual analog scale. The students scored their dogs for 20 different behaviors by drawing a mark perpendicular to the scale for each behavior in the first week of dog training (week 3, “PRE”) and the last week (week 14, “POST”).

Each student performed a QBA early and late in the semester using a form similar to the one pictured in Figure 2.

The preliminary behavior analysis was performed during week 3 of the dog socialization course (PRE) and the postliminary behavior analysis was performed during week 14 of the dog socialization course (POST). On the QBA score sheet, each behavior had a 125 mm visual analog scale ([41]; “line”) with the minimum being the leftmost end of the line (0 mm) and the maximum being the rightmost end of the line (125 mm). Students scored their dog by placing a vertical mark on the horizontal scale for each behavior. Data for the behaviors of Bored, Distressed, Friendly, Happy, Nervous, Positively Occupied, and Scared are missing for the Spring 2022 semester due to technical issues with the photocopier. The assessments were conducted live and on paper. During this specific semester a photocopying error occurred and the back page of the QBA tool was unintentionally omitted; thus, those students did not have access to the tool to assess Bored, Distressed, Friendly, Happy, Nervous, Positively Occupied, and Scared behaviors.

### 2.4. Data Analysis

Every student was assigned a numerical designation and every dog was assigned an alphabetical designation to maintain anonymity. Each student-made mark on the QBA sheets was measured for their distance from 0 and assigned a value between 0 and 125. The values were entered into an Excel spreadsheet with the identifying semester and year of the data as well as the identification designations of the dog and student that the data was collected from. Using this data, summary statistics were then calculated including average, standard error, and percent change between observation periods for each QBA metric. Records with missing or improperly labeled data points were omitted from the study. The interaction between semester and the period (PRE/POST) the dog was evaluated on QBA outcomes was evaluated using a Linear Mixed Model (PROC MIXED; SAS Institute Inc., Cary, NC, USA). The model included the number of times the dog participated in the socialization program as a covariate with a Satterthwaite approximation. The random effect included semester nested within the dog by student interaction. The model included a repeated effect of period with the dog by student interaction as the subject using compound symmetry. Differences between PRE and POST periods within semester were determined using least squared means with a Bonferroni Correction.

To evaluate how the different behaviors clustered and changed over time, a Principal Component Analysis was conducted. All variables were evaluated for collinearity and those with a Variance Inflation Factor (PROC REG) and their cumulative contribution to the overall variance (PROC FACTOR). None of the variables exhibited collinearity, all were retained in the model, and these variables were standardized (PROC STANDARD) prior to conducting the Principal Component Analysis (PROC PRINCOMP) with a varimax rotation. Principal components with eigenvalues greater than one were retained for final interpretation and for individual behaviors within a PC. Eigenvalues with absolute values greater than 0.300 were considered significant sources of variance.

## 3. Results

### 3.1. Socialization and Training Improved All 20 Behaviors Assessed

**Figure 3:**
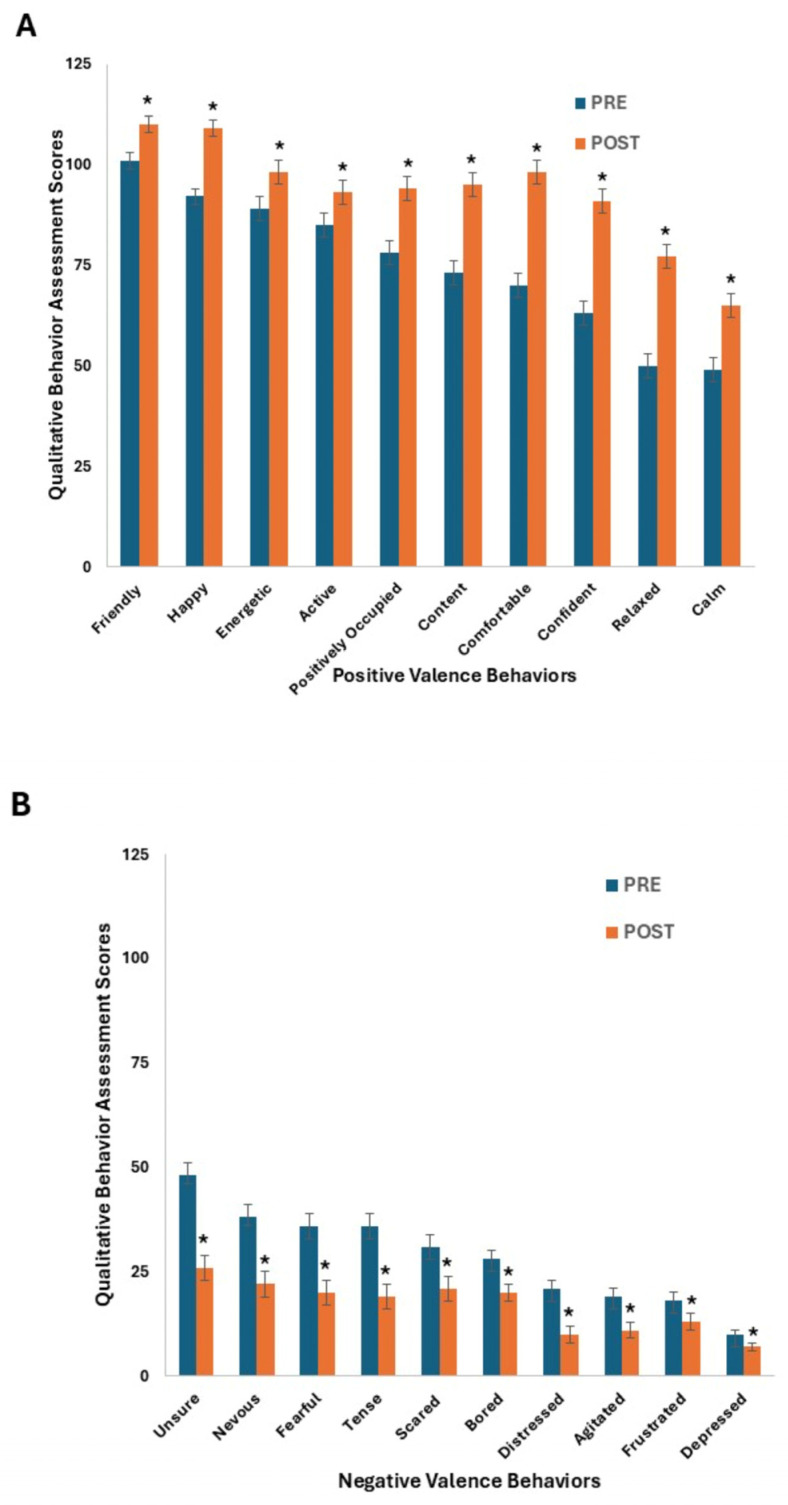
Qualitative assessments of 20 behaviors for “PRE” values (blue bars at left for each behavior) scored early in the semester and “POST” values (orange bars at right for each behavior) scored at the end of the semester are shown as least squares means and standard errors. The score for each behavioral term was recorded by measuring the distance in millimeters from the “minimum” anchor of the Visual Analog Scale (VAS). Each 125 mm VAS ranged from ‘Minimum’, indicating that the behavioral expression is entirely absent, to ‘Maximum’, meaning that the expressive quality is dominant. Panel (**A**). Dogs exhibited increases in positive valence behaviors over one semester of socialization and training. Panel (**B**). Dogs exhibited decreases in negative valence behaviors over the semester of socialization and training. In both panels (**A**,**B**), the y axis scale reflects the 125 mm lines that the students scored the behaviors on. The order of behaviors, from left to right, is based on the magnitude of the PRE values. In both panels (**A**,**B**), asterisks over POST values indicate significant differences from PRE values for that behavior (*p* < 0.05).

When QBA data from all nine semesters were considered together, both suites (i.e., positive and negative valence) of behaviors that the QBA metrics assessed at the end of the semester (POST) appeared to change in the desired direction from those assessed in the beginning (PRE; Figure 3 and Table S1).

A dog’s prior experience in participating with the socialization program did not influence any of the behaviors assessed. All dogs began the semesters exhibiting high levels of positive valence behaviors (mean ± SE: 75 ± 6 mm), including Happy, Active, and Comfortable (Figure 3A); and the ten positive valence behaviors improved (increased in their QBA scores) over the semester (*p* ≤ 0.006). On average, these ten behaviors increased 24% throughout the semester. The greatest changes in positive valence behaviors were observed in the qualities of Relaxed (53%), Confident (43%), and Comfortable (41%) and the smallest increase (9%) was for Friendly, which also had the highest mean in the PRE QBA data. Negative valence behavior scores were low at the beginning of class (31 ± 3 mm) and all these metrics improved (declined in their QBA scores) over the semesters (Figure 3B). Negative valence qualities decreased on average 42% (*p* < 0.005). The greatest decreases were observed for the qualities of Distressed (50%) and then Tense, Unsure, and Fearful (48 to 44%) and the smallest decreases (29%) were for Bored and Frustrated. There were consistent improvements or trends toward improvements when the QBA scores were analyzed within and across each semester (Figure S1). In the cases of Comfortable (Figure S1c), Confident (Figure S1f), Fearful (Figure S1k), Relaxed (Figure S1q), and Unsure (Figure S1t), the majority of individual semesters demonstrated improvements (*p* < 0.05). We believe the missing data from Spring 2022 for the seven behaviors (Bored, Distressed, Friendly, Happy, Nervous, Positively Occupied, and Scared) did not affect the data presented because the missing data represented a small fraction (<5%) of the total points assessed. Additionally, the data in the four previous and four subsequent semesters are consistent with each other for each behavior. Overall, while the behaviors of the dogs at the beginning of the semester were good, each one that we assessed appeared to increase or decrease as desired after participation in a semester-long socialization program.

### 3.2. Principal Component Analysis Identifies Dimensions Describing Variations in the Dogs’ Behavior Scores from PRE to POST Semester Data

Principal Component Analysis (PCA) identified groups of behaviors (PC1, PC2, PC3 and PC4) with eigenvalues (values of covariance) > 1.0 (Table 2).

**Figure 4:**
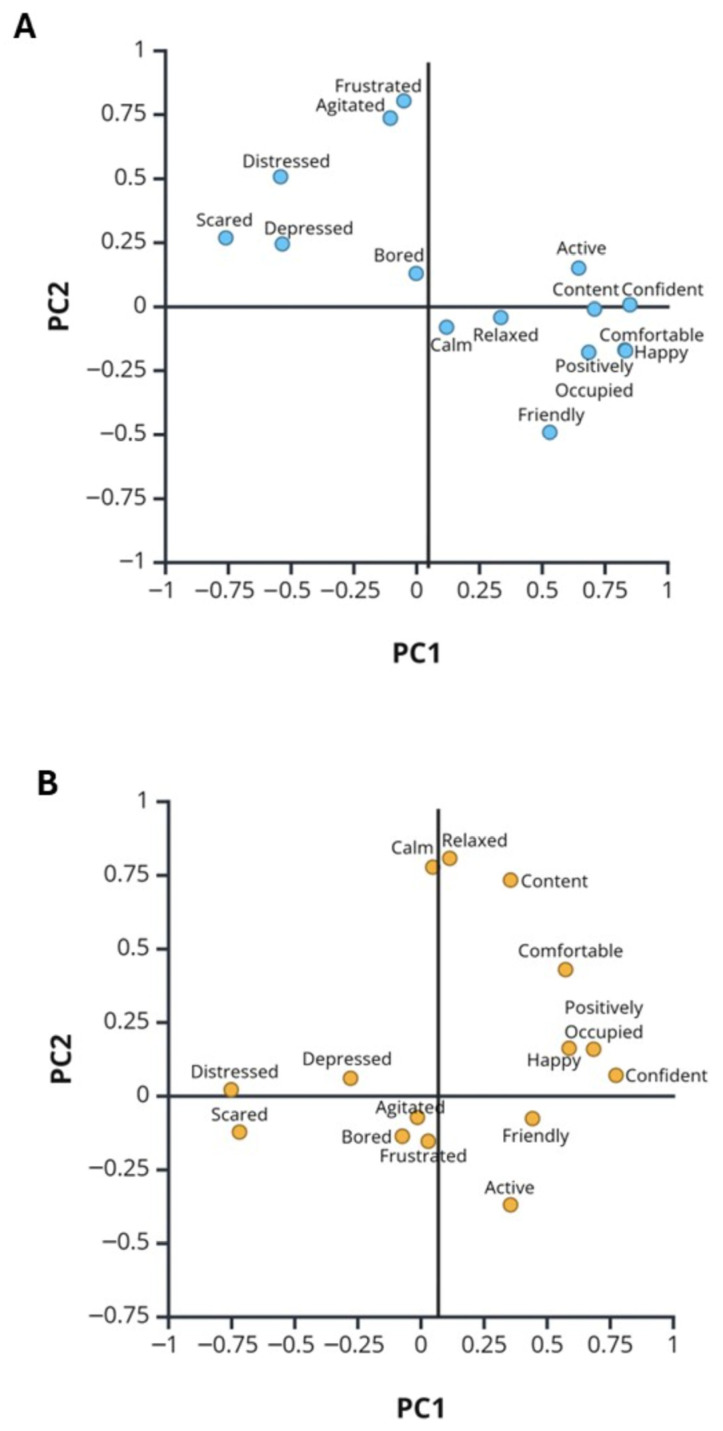
The distribution of QBA term loadings along the first two PCA components for the PRE data (panel (**A**), blue circles) and the POST data (panel (**B**), orange circles) are plotted. Circles labeled with more than one QBA metric are overlapping or very close for those two or more metrics. Created in BioRender. Ing, N. (2025) https://BioRender.com/vry7fho (accessed on 10 June 2025).

Eigenvalues for behaviors within a PC that were ≥0.300 or ≤−0.300 were considered significant and are in bold font. For the PRE QBA data, four components explained 69% of the variance observed. The first component represented a general size effect where several variables contribute equally, but moderately (Table 2A). The second component of the PRE data included Active, Calm, Energetic, and Relaxed, the third component included the behaviors Agitated, Bored, and Frustrated, and the fourth component was defined by the behaviors of Fearful, Happy, and Positively Occupied. For the POST QBA data, four components explained 62% of the variance observed. The first component included positive associations between the behaviors of Nervous and Tense (Table 2B). The second component included positive associations between the behaviors of Calm and Relaxed along with negative associations of Active and Energetic. The third post-socialization component was characterized by positive associations for Happy and Nervous along with a negative association with Frustrated. The fourth component was defined by the behaviors of Agitated, Content, Friendly, Frustrated, and Relaxed. PC1 and PC2 described the major sources of variance and the eigenvalues of the 20 behaviors were plotted against each other for PRE (Figure 4A) and POST (Figure 4B) QBA data.

The plots show that the variances in the QBA metrics were similar in PRE and POST periods. In both plots, the metrics that were related in operational definitions (Table 1) were clustered together. The two plots in Figure 4 indicate that the major sources of variance described by PC1 and PC2 in the PRE and POST data were not different.

## 4. Discussion

The welfare of dogs housed in kennels at universities is important to maximize the success of their respective research and teaching programs. Our large study illustrated improvements in dog behaviors from socialization and training by students, notwithstanding the inherent flaws of the current study (e.g., lack of evaluator blinding). Indeed, all 20 behaviors assessed changed (behavioral profiles of PRE and POST QBA scores appeared to be different) over the 12 weeks of training in ways that were desired. The dogs participating in the class had QBA outcomes characterized by increases in ten of the positive valence behaviors and decreases in ten of the negative valence behaviors, demonstrating a perceived improvement in their welfare state. Our results are consistent with other studies that observed that increased frequency of positive or neutral human interactions can have favorable effects on the behaviors of kenneled dogs [17,18,19,20,21]. While these results also demonstrate the capacity for a socialization program to benefit dogs after their critical windows of development have closed, we acknowledge that the evaluators were not blinded to the treatment, thus presenting the possibility that the QBA outcomes were unintentionally influenced by evaluator bias.

The impact could, arguably, have been greater had these animals been socialized as puppies and future studies should consider a way to blind the evaluator to the assessments (e.g., a video assessment of the animal decoded by a blinded observer). However, reality dictated that these animals enter into a socialization program later in life and the approach was exploratory. The receipt of these animals after maturity poses unique challenges regarding the individualized socialization strategy and expected outcome. This study suggests that even though each dog’s inherent challenge was unique, participation in a socialization program was associated with benefits to kenneled dog welfare.

Increased human interactions prepare kenneled dogs for their post-retirement lives in private homes, yet specific strategies for successfully socializing dogs to provide the best welfare remain to be defined. Towards that aim, students in the current study socialized and trained their assigned dogs for three 30 min sessions per week for 12 weeks. The socialization and training of shelter dogs for only 20 min three days a week for eight weeks improved their behavior [18]. Fifteen minutes of human interaction was as effective as 30 min in short-term reductions in anxious behavior and plasma cortisol levels in shelter dogs [20]. Research beagles showed improved social behaviors after intensive, positive handling by a human for only 30 s a day for two months [21]. While our study did not include a group of control dogs lacking socialization and training, the differences observed were repeatable across multiple semesters and multiple dog–student pairings. In addition, other studies that included such a group only identified improvements in the behaviors of dogs in the group that received increased human interactions during experimental periods that were similar to ours [18,21,42]. The dogs in our socialization and training class appeared to benefit from the socialization but also from the written training information that facilitated continuity of care by providing a plan for the dog’s future training by students, staff, and private adopters. Together, the enhanced behaviors and training plans present a strategy for retaining institutional knowledge for each animal across multiple caretakers, thus benefiting the current and future welfare of dogs [43].

Although the evaluation of management practices is usually part of animal welfare assessments, animal-based measures are also necessary to evaluate how the animals are coping with their current situation [11]. Animal-based measures can include behavioral, physiological, or cognitive components. Physiological assessments of stress, such as cortisol levels, are frequently used, but a realistic picture of an animal’s welfare requires a multidisciplinary approach [33]. Our study did not include any physiological measures, which could be done in future studies. Quality-of-life measurements as part of welfare assessments often include QBAs, and these are best done by people familiar with the animal. It is important that QBAs, which are subjective measures, are validated previously to correlate with Quantitative Behavior Analyses, which use data such as cortisol levels [29]. The use of QBAs has been validated in numerous animals including pigs, cattle, horses, sheep, goats, and dogs [28,41,44,45,46,47]. The QBA used in this study [37] was chosen because it had been validated previously [38,39].

Several factors can influence how a student evaluates a dog using the QBA. Those factors include their educational background, experience with animals and dog behavior in particular, emotional mindset, and fatigue, as well as the type of activity around the dog during the assessment, and the accuracy of correctly identifying the defined behavior [48,49]. Students bring diverse backgrounds, knowledge, and experience into an observation setting which will influence how they perceive the dog’s behavior at the beginning and end of each semester. Although observer bias may influence the scores given by an individual, the general improvements noted in all the QBA scores are trends over nine semesters from 103 different individuals. Furthermore, the students received little formal training in the QBA. This paradigm mirrors what would be found in a local shelter or small animal veterinary clinic or kennel. Even without extensive training, differences were able to be observed and the tool was effective in characterizing the dogs’ overall countenance. This dataset is large and robust, thus any differences detected may not be influenced by the amount of training the observers received prior to data collection. The improvements in the dogs’ behaviors in the QBAs were also noted subjectively and independently by the instructor, the two professional trainers involved in the class, and the care staff working with the dogs.

Principal Component Analysis identified four dimensions for the 20 QBA behaviors across the dogs in PRE and then POST semesters. Our PC1 versus PC2 score plots of the PRE and POST QBA data were very similar to score plots in other studies that used QBAs with 20 terms to score shelter dogs in Italy [40] and Hungary [31,32]. Our 20 QBA behavior terms had 11 and 14 that were the same as or very similar to the QBA terms used by those groups, respectively. A difference in PC2 in both European studies compared to ours was that the shelter dogs were less relaxed than our dogs were in PRE and POST observation periods, perhaps due to the latter being acclimated to their environment. The differences in PC3 and PC4 eigenvalues observed between the PRE and the POST QBAs may be related to altered activation of some of the seven basic emotional systems critical to survival that were identified by Jaak Panksepp and are linked to subcortical regions of the mammalian brain [50]. For example, the decreases in Distressed, Frustrated, and Agitated may be related to lowered activity of the RAGE/anger system, while the increases in Calm, Relaxed, Content, and Comfortable may indicate up-regulation of the CARE/nurturing system. Repeated positive interactions with a human may have desensitized our dogs to human interactions and provided them the opportunity to build a human–animal connection. Panksepp’s creation of the field of Affective Neuroscience may help us understand dog behavior and be more able to help those with problems.

A tangential outcome of the project was recognition of how much students in the class benefited from the experience. Students were uniformly surprised by how much they learned from their high-impact learning experiences with the dogs. The students and dogs formed strong bonds within a few weeks. Students learned to train animals, a lifelong skill useful with most animal species. While one goal of the class was to reduce stress in the dogs, an additional benefit was that student stress was relieved by interacting with their dogs. Indeed, 60% of college students in the United States self-report experiencing acute and/or chronic stress [51]. A quantitative study of animal interactions with college students for an average of 26 min around the time of final exams positively impacted the students’ mood states [52]. A systematic review of young people experiencing animal-assisted interactions, activities, or therapies noted that sessions of >15 min decreased their levels of salivary cortisol [53]. These studies have led the way to numerous campuses, including Texas A&M University in College Station, Texas, offering students free sessions with therapy dogs during times of midterm and final exams. Although we did not directly assess students’ stress levels, there was anecdotal and self-reported evidence that the students in the Dog Socialization and Training class benefited similarly from interacting with their dogs.

The Dog Socialization and Training class continues to benefit Texas A&M University and similar programs could be expanded in the future. This class was very cost effective: students paid for their one credit hour of class each semester, while the Comparative Medicine Program paid the professional dog trainers and for the needed equipment and supplies. The class could easily be adopted by other universities and expanded to include more species. Other organizations including animal shelters and youth groups could adopt equivalent programs as well. The need continues for more of these activities that improve the lives of animals while simultaneously benefiting the lives of people.

## 5. Conclusions

For many reasons, puppies around the globe may not grow up to adulthood in a caring, private home. For those dogs that are institutionalized, their lack of early socialization can result in the establishment of fear-based behaviors. Our study demonstrates that a modest amount of human interaction with kennel-raised adult dogs, even after critical periods of development, has the potential to impact their behaviors within a 12-week period. By the conclusions of the semesters, the dogs were perceived to be adjusted, happier, more relaxed, and less nervous. However, a rigorous evaluation of how, specifically, to realistically implement and optimize a socialization program is needed. Suggestions for enhancing the experimental design of future studies are discussed above. Implementing a socialization program for kenneled dogs has the potential to improve their long-term welfare while simultaneously improving teaching and research outcomes, as well as helping the dogs cope with the adoption process and adapt to their future home environments.

## Figures and Tables

**Table 1:** Operational definitions of the metrics evaluated in the Qualitative Behavior Analyses (QBAs) [30,31,40].

QBA Metric	Operational Definition
Active	Animated, attracted to stimuli, explorative, energetic
Agitated	Unable to settle or cope with environment, impatient, restless
Bored	Uninterested, inactive, passive
Calm	Carefree, easy going, not tense
Comfortable	Without worries, settled in the environment
Confident	Interested in the environment and new stimuli, investigative
Content	Satisfied, doing positive activities, relaxed
Depressed	Dull, apathetic, disengaged from the environment
Distressed	Worried, apprehensive, tense
Energetic	Insistent, interactive, animated
Fearful	Timid, scared, afraid
Friendly	Seeks contact/interaction, affectionate
Frustrated	Conflict behavior, uneasy, stressed
Happy	Cheerful, engaging in and inviting others to play
Nervous	Uneasy, unsure, agitated
Positively Occupied	Exploring, attentive, interested
Relaxed	Interested in surroundings, not nervous or tense
Scared	Fearful, nervous, unsure
Tense	Uneasy, cautious, suspicious
Unsure	Reluctant, withdrawn, worried

**Table 2:** Principal Component Analysis (PCA) identified four Principal Components (PC1, PC2, PC3, and PC4) and loadings of Qualitative Behavior Analysis (QBA) terms within these factors. Panel (**A**) is from the PRE QBA data, while panel (**B**) is from the POST QBA data. Eigenvalues for behaviors within a PC with absolute values ≥ 0.300 are in bold.

**(A)**				
**QBA Metric**	**PC1**	**PC2**	**PC3**	**PC4**
Active	−0.206	**−0.392**	0.056	0.274
Agitated	0.122	−0.232	**0.448**	0.051
Bored	0.051	0.046	**0.444**	−0.233
Calm	0.048	**0.552**	0.148	0.000
Comfortable	−0.277	0.154	0.183	0.105
Confident	−0.277	−0.056	0.204	−0.014
Content	−0.219	0.177	0.235	0.258
Depressed	0.224	0.105	0.237	−0.052
Distressed	0.230	−0.063	0.165	0.090
Energetic	−0.229	**−0.333**	0.128	0.284
Fearful	0.260	−0.036	0.019	**0.393**
Friendly	−0.210	0.181	−0.172	0.096
Frustrated	0.083	−0.221	**0.479**	−0.235
Happy	−0.272	0.026	0.098	**0.319**
Nervous	0.287	−0.031	−0.056	0.293
Positively Occupied	−0.223	0.121	0.045	**0.306**
Relaxed	−0.100	**0.436**	0.266	0.085
Scared	0.297	0.033	0.015	0.276
Tense	0.286	0.074	0.052	0.264
Unsure	0.277	0.076	0.010	0.213
PC eigenvalue	8.429	2.337	1.552	1.454
Variance explained (%)	42.2%	11.7%	7.8%	7.3%
**(B)**				
**QBA Metric**	**PC1**	**PC2**	**PC3**	**PC4**
Active	−0.125	**−0.463**	0.289	0.177
Agitated	0.107	−0.158	−0.212	**0.482**
Bored	0.129	0.087	−0.254	−0.065
Calm	−0.033	**0.489**	0.128	0.144
Comfortable	−0.251	0.255	0.090	−0.003
Confident	−0.272	−0.090	0.049	0.164
Content	−0.208	0.269	0.182	**0.364**
Depressed	0.156	0.129	−0.101	0.072
Distressed	0.297	0.070	0.125	0.097
Energetic	−0.162	**−0.450**	0.223	0.142
Fearful	0.249	0.019	0.281	0.204
Friendly	−0.177	0.083	0.146	**−0.383**
Frustrated	0.117	−0.120	**−0.380**	**0.327**
Happy	−0.261	0.041	**0.329**	−0.059
Nervous	**0.320**	−0.026	**0.303**	−0.034
Positively Occupied	−0.282	−0.060	0.153	0.129
Relaxed	−0.115	**0.338**	0.083	**0.420**
Scared	0.290	0.031	0.292	−0.061
Tense	**0.316**	−0.022	0.246	0.135
Unsure	0.287	0.029	0.224	−0.056
PC eigenvalue	6.64	2.65	1.79	1.39
Variance explained (%)	33.2%	13.3%	9.0%	6.9%

## Data Availability

The original contributions presented in this study are included in the article/Supplementary Materials. Further inquiries can be directed to the corresponding author.

## Supplementary Materials

The following supporting information can be downloaded at: https://www.mdpi.com/article/10.3390/ani16030485/s1, Figure S1: Qualitative Behavior Assessment (QBA) scores for the 20 behaviors at the beginning and end of each semester (means ± SEs) are graphed; Table S1: Qualitative Behavior Analyses metrics improved from the beginning (PRE) to the end (POST) of the semesters.

## Abbreviations

The following abbreviations are used in this manuscript:

IACUC	Institutional Animal Care and Use Committee
PCA	Principal Component Analysis
POST	At the end of the semester
PRE	At the beginning of the semester
QBA	Qualitative Behavior Assessment
TDCJ	Texas Department of Criminal Justice
VAS	Visual Analog Scale

## References

[1] Animal and Plant Health Inspection Service FY2023 Research Animal Use Summary. https://www.aphis.usda.gov/sites/default/files/fy2023-research-animal-use-summary.pdf.

[2] Switonski M. (2014). Dog as a model in studies on human hereditary diseases and their gene therapy. Reprod. Biol..

[3] McGreevy J.W., Hakim C.H., McIntosh M.A., Duan D. (2015). Animal models of Duchenne muscular dystrophy: From basic mechanisms to gene therapy. Dis. Models Mech..

[4] Adin C.A., Gilor C. (2017). The diabetic dog as a translational model for human islet transplantation. Yale J. Biol. Med..

[5] Shepherd C., Wangchuk P., Loukas A. (2018). Of dogs and hookworms: Man’s best friend and his parasites as a model for translational biomedical research. Parasites Vectors.

[6] Kruitwagen H.S., Penning L.C. (2019). Preclinical models of Wilson’s disease, why dogs are catchy alternatives. Ann. Transl. Med..

[7] Barthelemy I., Hitte C., Tiret L. (2019). The dog model in the spotlight: Legacy of a trustful cooperation. J. Neuromus. Dis..

[8] Zhu W., Wu J., Zhao H., Wang W., Lu L., Yan K., Yin Y., Huang Q. (2020). Establishment and characteristic analysis of a dog model for autologous homologous cranioplasty. Biomed. Res. Int..

[9] Hanlon A., Gath V., Mulligan F. (2007). Practical animal-handling classes at University College Dublin. J. Vet. Med. Educ..

[10] Cawdell-Smith A.J., Pym R.A., Verrall R.G., Hohenhaus M.A., Tribe A., Coleman G.T., Bryden W.L. (2007). Animal handling as an integrated component of animal and veterinary science programs at the University of Queensland. J. Vet. Med. Educ..

[11] Polgar Z., Blackwell E.J., Rooney N.J. (2019). Assessing the welfare of kennelled dogs—A review of animal-based measures. Appl. Anim. Behav. Sci..

[12] Beaver B.V. (2009). Canine social behavior. Canine Behavior: Insights and Answers.

[13] McMillan F.D., Duffy D.L., Serpell J.A. (2011). Mental health of dogs formerly used as ‘breeding stock’ in commercial breeding establishments. Appl. Anim. Behav. Sci..

[14] Serpell J.A., Duffy D.L., Jagoe J.A. (2017). Becoming a dog: Early experience and the development of behavior. The Domestic Dog: Its Evolution, Behavior, and Interactions with People.

[15] Romaniuk A.C., Barnard S., Shreyer T., Croney C. (2025). Effects of dam fear and stress on metrics of puppy welfare in commercial breeding kennels. Sci. Rep..

[16] Buttner A.P., Awalt S.L., Strasser R. (2023). Early life adversity in dogs produces altered physiological and behavioral responses during a social stress-buffering paradigm. J. Exp. Anal. Behav..

[17] Wells D.L. (2004). A review of environmental enrichment for kennelled dogs, *Canis familiaris*. Appl. Anim. Behav. Sci..

[18] Bergamasco L., Osella M.C., Savarino P., Larosa G., Ozella L., Manassero M., Badino P., Odore R., Barbero R., Re G. (2010). Heart rate variability and saliva cortisol assessment in shelter dogs: Human-animal interaction effects. Appl. Anim. Behav. Sci..

[19] Cafazzo S., Maragliano L., Bonanni R., Scholl F., Guarducci M., Scarcella R., Di Paolo M., Pontier D., Lai O., Carlevaro F. (2014). Behavioral and physiological indicators of shelter dogs’ welfare: Reflections on the no kill policy in Italy revisited on the basis of 15 years of implementation. Physiol. Behav..

[20] Willen R.M., Mutwill A., MacDonald L.J., Schiml P.A., Hennessy M.B. (2017). Factors determining the effects of human interaction on the cortisol levels of shelter dogs. Appl. Anim. Behav. Sci..

[21] Hubrecht R.C. (1993). A comparison of social and environmental enrichment methods for laboratory housed dogs. Appl. Anim. Behav. Sci..

[22] Conley M.J., Fisher A.D., Hemsworth P.H. (2014). Effects of human contact and toys on the fear responses to humans of shelter-housed dogs. Appl. Anim. Behav. Sci..

[23] Van der Laan J.E., Vinke C.M., Arndt S.S. (2022). Evaluation of hair cortisol as an indicator of long-term stress responses in dogs in an animal shelter and after subsequent adoption. Sci. Rep..

[24] Roth L.S.V., Faresjo A., Theodorsson E., Jensen P. (2016). Hair cortisol varies with season and lifestyle and relates to human interactions in German shepherd dogs. Sci. Rep..

[25] Stella J., Shreyer T., Ha J., Croney C. (2019). Improving canine welfare in commercial breeding operations: Evaluating rehoming candidates. Appl. Anim. Behav. Sci..

[26] Wemelsfelder F., Hunter T.E., Mendl M.T., Lawrence A.B. (2000). The spontaneous qualitative assessment of behavioural expressions in pigs: First explorations of a novel methodology for integrative animal welfare measurement. Appl. Anim. Behav. Sci..

[27] Wemelsfelder F., Hunter T.E., Mendl M.T., Lawrence A.B. (2001). Assessing the ‘whole animal’: A free choice profiling approach. Anim. Behav..

[28] Rousing T., Wemelsfelder F. (2006). Qualitative assessment of social behaviour of dairy cows housed in loose housing systems. Appl. Anim. Behav. Sci..

[29] Walker J.K., Dale A.R., D’Eath R.B., Wemelsfelder F. (2016). Qualitative Behaviour Assessment of dogs in the shelter and home environment and relationship with quantitative behaviour assessment and physiological responses. Appl. Anim. Behav. Sci..

[30] Minero M., Costa E.D., Dai F., Canali E., Barbieri S., Zanella A., Pascuzzo R., Wemelsfelder F. (2018). Using qualitative behaviour assessment (QBA) to explore the emotional state of horses and its association with human-animal relationship. Appl. Anim. Behav. Sci..

[31] Stubjøen S.M., Oppermann Moe R., Bruland K., Lien T., Muri K. (2020). Reliability of observer ratings: Qualitative behaviour assessments of shelter dogs using a fixed list of descriptors. Vet. Anim. Sci..

[32] Stubjøen S.M., Oppermann Moe R., Johannessen C., Larsen M., Madsen H., Muri K. (2022). Can shelter dog observers score behaviour expressions consistently over time?. Acta Vet. Scand..

[33] Lamon T.K., Slater M.R., Moberly H.K., Budke C.M. (2021). Welfare and quality of life assessments for shelter dogs: A scoping review. Appl. Anim. Behav. Sci..

[34] American Psychological Association Dictionary of Psychology. https://dictionary.apa.org.

[35] Juge A.E., Hall N.J., Richeson J.T., Daigle C.L. (2022). Using canine olfaction to detect bovine respiratory disease: A pilot study. Front. Vet. Sci..

[36] Johnson A.C., Wynne C.D.L. (2024). Comparing the efficacy in reducing pulling and welfare impacts of four types of leash walking equipment. PeerJ.

[37] Welfare Quality^®^ (2009). Welfare Quality^®^ Assessment Protocol for Poultry (Broilers, Laying Hens).

[38] Bassler A.W., Arnould C., Butterworth A., Colin L., DeJong C., Ferrante V., Ferrari P., Haslam S., Wemelsfelder F., Blokhuis H.J. (2013). Potential risk factors associated with contact dermatitis, lameness, negative emotional state, and fear of humans in broiler chicken flocks. Poult. Sci..

[39] Muri K., Stubjøen S.M., Vasdal G., Moe R.O., Granquist E.G. (2019). Associations between qualitative behaviour assessments and measures of leg health, fear, and mortality in Norwegian broiler chicken flocks. Appl. Anim. Behav. Sci..

[40] Arena L., Wemelsfelder F., Messori S., Ferri N., Barnard S. (2019). Development of a fixed list of terms for the Qualitative Behavioural Assessment of shelter dogs. PLoS ONE.

[41] Arena L., Wemelsfelder F., Messori S., Ferri N., Barnard S. (2017). Application of free choice profiling to assess the emotional state of dogs housed in shelter environments. Appl. Anim. Behav. Sci..

[42] Kiddie J.L., Collins L.M. (2015). Identifying environmental and management factors that may be associated with the quality of life of kennelled dogs (*Canis familiaris*). Appl. Anim. Behav. Sci..

[43] Rooney N., Stafford K., Yeates J. (2019). Dogs (*Canis familiaris*). Companion Animal Care and Welfare: The UFAW Companion Animal Handbook.

[44] Rutherford K.M.D., Donald R.D., Lawrence A.B., Wemelsfelder F. (2012). Qualitative Behaviour Assessment of emotionality in pigs. Appl. Anim. Behav. Sci..

[45] Minero M., Tosi M.V., Canali E., Wemelsfelder F. (2009). Quantitative and qualitative assessment of the response of foals to the presence of an unfamiliar human. Appl. Anim. Behav. Sci..

[46] Wickham S.L., Collins T., Barnes A.L., Miller D.W., Beatty D.T., Stockman C., Blache D., Wemelsfelder F., Fleming P.A. (2012). Qualitative behavioral assessment of transport-naïve and transport-habituated sheep. J. Anim. Sci..

[47] Grosso L., Battini M., Wemelsfelder F., Barbieri S., Minero M., Costa E.D., Mattiello S. (2016). On-farm Qualitative Behaviour Assessment of dairy goats in different housing conditions. Appl. Anim. Behav. Sci..

[48] Kiddie J.L., Collins L.M. (2014). Development and validation of a quality of life assessment tool for use in kennelled dogs (*Canis familiaris*). Appl. Anim. Behav. Sci..

[49] Martin P., Bateson P. (2007). Measuring Behaviour: An Introductory Guide.

[50] Davis K.L., Montag C. (2019). Selected principles of Pankseppian affective neuroscience. Front. Neurosci..

[51] Flaherty C. Stress is Hurting College Students. https://www.insidehighered.com/news/student-success/health-wellness/2023/05/23/survey-stress-hurting-college-students.

[52] Robino A.E., Corrigan V.K., Anderson B., Were S., Farley J.P., Marmagas S.W., Buechner-Maxwell V. (2021). College student mental health in animal-assisted intervention program: A preliminary study. J. Creat. Ment. Health.

[53] Pena-Jorquera H., Hernandez-Jana S., Sanchez-Martinez J., Espinoza-Puelles J.P., Martínez-Flores R., Barreto-Schuch F., Yáñez-Sepúlveda R., Delgado-Floody P., Ferrari G., Sadarangani K.P. (2025). Dog companionship and cortisol levels in youth: A systematic review and meta-analysis. Soc. Sci. Med..

