# Supplementary material for: Participation in a Short-Term Socialization and Training Program Improved Kennel-Raised Dog Welfare
Source: Animals (Basel). 2026 Feb 4;16(3):485. doi: 10.3390/ani16030485 (PMC12897358; doi:10.3390/ani16030485)
Supplement: Supplementary file 1 [file animals-16-00485-s001.zip › IngTableS1_121125.pdf]

Table S1. Qualitative Behavior Analyses metrics improved from the beginning (PRE) to the end (POST) of the semesters. Qualitative Behaviour Analysis (QBA) metrics were evaluated in kennel-housed dogs at the beginning (PRE) and end (POST) of participation in a 12-week socialization and training program. Students scored their dogs for each of the 20 behaviors on 125 mm-long visual analog scales, which generated values from 0 to 125. Comparisons between QBA scores observed during PRE were compared to those collected at POST using a Linear Mixed Model and the percentage difference between the two was calculated (% change). Means (PRE,POST) and standard errors of the means (SEM) are presented.

QBA Metric	PRE	POST	SEM	P-value	% change
Active	84.54	93.21	2.64	0.006	10%
Agitated	18.89	11.42	1.63	<0.0001	-40%
Bored	28.42	20.21	2.44	0.0011	-29%
Calm	49.09	64.47	2.78	<0.0001	31%
Comfortable	69.62	98.11	2.58	<0.0001	41%
Confident	63.23	90.60	2.50	<0.0001	43%
Content	72.89	95.30	2.46	<0.0001	31%
Depressed	10.10	6.58	1.23	0.0049	-35%
Distressed	21.19	10.52	2.07	<0.0001	-50%
Energetic	89.42	98.41	2.86	0.0021	10%
Fearful	36.12	20.23	2.47	<0.0001	-44%
Friendly	100.77	110.17	2.01	<0.0001	9%
Frustrated	18.42	13.14	1.77	<0.0001	-29%
Happy	92.20	108.97	2.21	<0.0001	15%
Nervous	38.14	22.36	2.54	<0.0001	-41%
Positively Occupied	78.15	93.86	2.94	<0.0001	20%
Relaxed	50.30	76.94	2.57	<0.0001	53%
Scared	31.50	20.98	2.73	0.0002	-33%
Tense	36.28	18.69	2.58	<0.0001	-48%
Unsure	48.03	26.48	2.80	<0.0001	-45%
