# Supplementary material for: Participation in a Short-Term Socialization and Training Program Improved Kennel-Raised Dog Welfare
Source: Animals (Basel). 2026 Feb 4;16(3):485. doi: 10.3390/ani16030485 (PMC12897358; doi:10.3390/ani16030485)
Supplement: Supplementary file 1 [file animals-16-00485-s001.zip › IngFigS1_121225.pdf]

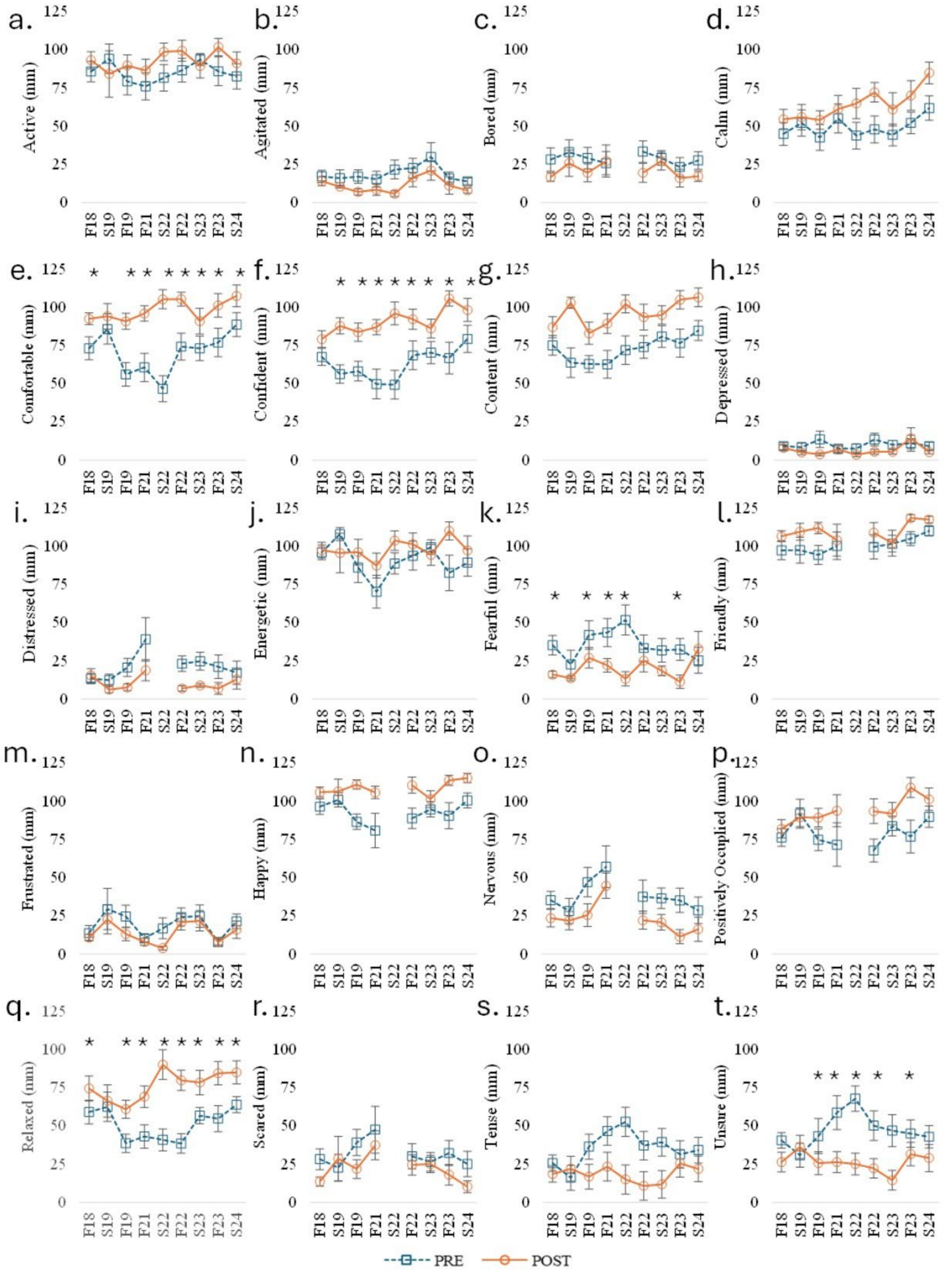

Figure S1. Qualitative Behavior Assessment (QBA) scores for the 20 behaviors at the beginning and ends of each semesters (means ± SEs) are graphed. QBAs were conducted on kennel-housed dogs prior to (PRE; blue □) and at the end (POST; orange ○) of participating in a semester-long socialization program. The score for each behavioral term was recorded by measuring the distance in millimeters from the "minimum" anchor of the Visual Analog Scale (VAS).

Each 125 mm VAS ranged from 'Minimum', indicating that the behavioral expression is entirely absent, to 'Maximum', meaning that the expressive quality is dominant. The behavioral expressions used were (in alphabetical order): a) Active, b) Agitated, c) Bored, d) Calm, e) Comfortable, f) Confident, g) Content, h) Depressed, i) Distressed, j) Energetic, k) Fearful, l) Friendly, m) Frustrated, n) Happy, o) Nervous, p) Positively Occupied, q) Relaxed, r) Scared, s) Tense, t) Unsure. Results (means ± SEs) are graphed for each metric in each semester with "*" indicating differences ($P < 0.05$) between PRE and POST scores within a semester. Note that summary data for the 20 metrics analyzed across all nine semesters are in Figure 3 and Table S1.
